# 

**Published:** 1891-12-26

**Authors:** 


PRICE ONE PENNY.

o. 274, Vol. XI.—December 26th, 1891.

[Registered at the General Post Office as a Newspaper for transmission in the United Kingdom and Abroad.]

WITH EXTRA NURSING SUPPLEMENT.
Per Annum, 6s. 6d., post free.
Monthly Parts, 6d.; post free, 8d.
Ditto per Annum, 8s., post free.

### THE HOSPITAL

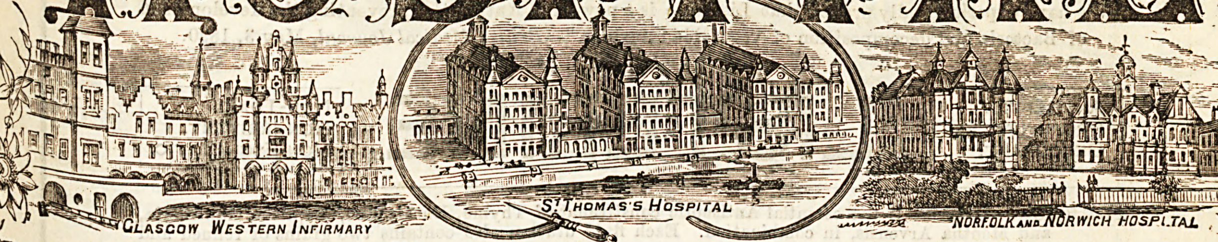

A WEEKLY JOURNAL OF

### Science, Medicine, Nursing, and Philanthropy.

SATURDAY, DECEMBER 26th, 1891.

Is it English? - 149

Popular Journalism - 150

Some Scientific Aspects of Insanity. VII.—Melancholia 151

The Story of the Insane from Year to Year - 152

Letters from Far Japan. By Mrs. Ernest Hart (*concluded*) 153

Everybody's Page - 153

Notes and News - 154

Passing Topics.—The Hospital Sunday Fund - 155

Scraps and Gleanings - 155

Annotations.—Gold too Dear—Eucalyptus for Fevers—Miss F. P. Cobbe on Health and Holiness - 156

The Practitioner's Mirror and Retrospect—

Medicine.—Cactus in Functional Affections of the Heart - 157

Surgery.—Papillomatous Ovarian Growths - 157

Ophthalmology.—Opacities of the Cornea - 158

New Drugs, Appliances, and Things Medical—Vaccine Case—Cellular Cloth Clothing—Anti-Rheumatic Towels—Knitted Corsets—Lobeck's Pure Soluble Cocoa—Coca Cocoa 159

Editor's Letter Box.—Hospital Reform - 160

Hospital Saturday Fund - 160

The Student of Medicine.—Appointments—Vacancies—Pass Lists.

EXTRA SUPPLEMENT.—THE NURSING MIRROR.

En Passant.—Short Items—Quiet Courage—A Canadian School—A Happy Christmas—Scottish Queen's Nurses—Australian Notes lxxiii

Lectures on Surgical Ward Work and Nursing. By Alex. Miles, M.D.Edin., C.M., F.R.C.S.E.—Lecture XL.—Urethral Instruments lxxiv

Christmas Competitions lxxv

For Reading to the Sick—A Merry Christmas! - lxxv

A Nurse in Natal (*continued*) - lxxvi

A Christmas Thought - lxxvi

Everybody's Opinion.—Nursing at the London Hospital—Glasgow Royal Infirmary—Local High Temperature—Boots and Shoes - lxxvii

Appointments - lxxvii

Presentations - lxxvii

Notes and Queries - lxxvii

Off Duty.—The Message of the Chimes - lxxviii

Amusements and Relaxation - lxxviii

